# The Healing Effect of Silicone Gel on Sciatic Nerve Injuries in Experimental Rat

**Published:** 2014-07

**Authors:** Hamid Reza Fathi, Mahdi Fathi, Alireza Ghannadan, Mina Alavion, Kambiz Kamyab, Zahra Khazaipour, Saeed Amanpour

**Affiliations:** 1 Plastic and Reconstructive Surgery Department, Vali-e-Asr Hospital, Tehran University of Medical Sciences, Tehran, Iran;; 2 Pathology Department, Razi Hospital, Tehran University of Medical Sciences, Tehran, Iran;; 3 Neurology Research Center, Tehran University of Medical Sciences, Tehran, Iran;; 4 Experimental Research Center, Institute Cancer, Tehran University of Medical Sciences, Tehran, Iran

**Keywords:** Silicon, Sciatic, Axon regeneration, Neuroma, Nerve Repair, Scar Formation

## Abstract

**BACKGROUND:**

Peripheral nerve repair is often complicated by fibroblastic scar formation, nerve dysfunction, and traumatic neuroma formation. Use of silicone may improve outcomes of these repairs. In this study, we tried to evaluate effectiveness of silicone gel on rats’ sciatic nerve repair, axon regeneration and scar formation around and in the nervous tissues.

**METHODS:**

This experimental study was performed on 18 rats. They underwent bilateral sciatic nerve dissection. Then, right and left damaged sciatic nerves were sutured. In left side, silicone gel was applied. Two months later, both sides were evaluated regarding to myelin fiber diameter (µm), total fascicular area (mm^2^), axon diameter (µm), myelin thickness (µm), G- ratio (axon diameter/myelin thickness), connective tissue area, ratio of connective tissue area/fascicular area, neuroma and foreign body formation in liver and lungs and spleen reaction. Results of right and left sides were compared.

**RESULTS:**

Silicone was significantly more effective in increasing myelin thickness in the side that silicone was applied) than the control side. It was not associated with inflammation, scar formation, granuloma, and neuroma formation. No foreign body reaction occurred in liver, spleen and lungs after silicone application; but axonal regeneration did not improve with after its use.

**CONCLUSION:**

According to our findings, it seems that silicone application in the cases with significant complications or in the cases that nerve graft is not possible would be an ideal option.

## INTRODUCTION

Reconstruction of damaged peripheral nerves is in association to connective tissue proliferation and scar formation in the nerve and adjacent tissues.^1^^-^^4^ It may result into neuroma formation, disturbance in axon regeneration and finally nerve dysfunction. Fibroblasts play an important role in nerve repair because they are essential components in epineurium, perineurium and endoneurium construction. Nevertheless, inappropriate and accelerated response of fibroblasts may be resulted in scar formation and axon regeneration disturbance.^5^^-^^9^

Although numerous studies have been yet performed on repairing nervous defect and developing an appropriate conduit for nerve growth, but just few investigations have been done on effectiveness of different materials on fibroblasts function, collagen production and scar formation.^1^^-^^7^^,^^10^^-^^13^

In some of these studies, various materials such as muscle, fat tissue, amnion, vein have been used as biologic barriers or absolvable materials such as collagen and polyglycolic have been applied as conduit.^5^^-^^7^^,^^9^^,^^10^^,^^12^^,^^14^^-^^18^ Although most of them were effective in nerve repair, some adverse effects have been reported. For example, using muscle and vein has been related to donor-site morbidity. In addition, these materials are not effective on collagen remodeling and fibroblasts function.^16^^,^^19^^-^^20^

Silicone is a safe and effective material with application in treatment of cutaneous scars. It has been applied in nerve repair as conduit and was shown to directly affect on fibroblasts and lead to an increase in temperature about 1 degree centigrade. It was demonstrated to improve the hydration and had a good effect on collagens kinetic. It can cause changes in expression of adhesion molecule of lymphatic infiltration and would be effective in collagen remodeling.^16^^,^^17^^,^^21^^,^^22^

According to silicone (polydimethyl siloxane) characteristics, we decided to evaluate effectiveness of silicone on nerve reconstruction, axon regeneration and scar formation around and in nervous tissues.

## MATERIAL AND METHODS

In an experimental study, Wistar male rats were provided from Laboratory Animal Center of Tehran University of Medical Sciences (TUMS). Ethical consideration of this study have been evaluated and approved by TUMS Ethical Committee (code: 1347). All rats were reserved in similar nutritional, temperature and environmental conditions. 

At first, rats underwent anesthesia by intraperitoneal injection of a combination of ketamine (80 mg/kg) and xylazine (5 mg/kg). Then, in the prone position, surgical site was disinfected with povidine iodine and shaved. After that, right and left side sciatic nerve were exposed with a 2 cm incision in the proximal area of thigh. In the 1.5 cm distance from sciatic foramen, sciatic verve was sharply dissected from adjacent tissues and cut with microsurgery scissors. Then, it was repaired with 3-4 sutures (nylon 9.0) under loop magnification. At the left side, silicone gel [Spectragel (Synfix) with effective material as polysiloxane, USA) was used so that anastomosis site as well as its distal and proximal areas were immersed in gel. Finally, the skin was closed with nylone 2.0.

Two months later, Anastomosis sites were evaluated regarding histological criteria such as scar formation and axon regeneration (major outcomes), neuroma formation and complications such as foreign body reaction (minor outcomes). In addition, possible systemic effects of silicone gel were evaluated by assessing liver, spleen and lung samples. So a sample from left lobe of liver, from spleen and from inferior lobe of right lung were obtained and sent to pathological laboratory in 10% formalin buffer. These samples were further stained with hematoxilin and eosin (H&E) for histological evaluation. 

Sciatic nerves were evaluated regarding to myelin fiber diameter (µm), total fascicular area (mm^2^), axon diameter (µm), myelin thickness (µm), G-ratio (axon diameter/myelin thickness), connective tissue area, ratio of connective tissue area/fascicular area, foreign body reaction, neuroma formation and foreign body formation in liver, lungs and spleen.

At first, incision site was re-incised and grossly observed regarding to adhesion in adjacent tissues and neuroma formation. Based on adhesion, they were classified in three grades of (i) Grade 1: no or mild blunt dissection, (ii) Grade 2: vigorous blunt dissection and (iii) Grade 3: sharp dissect. Thereafter, sciatic nerve was evaluated in the anastomosis site regarding possible disruption; and then paraffin embedded nerve samples were stained with H&E and Gomori trichrome staining. Nerve area and diameter were measured by Olympus DP20 camera.

Data were analyzed by SPSS software (Version 11, Chicago, IL, USA). We used KS method for evaluating distribution of quantitative data. P-pair test as a parametric test was used when distribution was normal; whereas, Wilcoxon signed rank test was used for data without normal distribution. McNemar test was used for comparing qualitative variables .P-value less than 0.05 was considered as significance level.

## RESULTS

Two out of 20 rats were excluded from the study because of missing data about them. So sciatic nerves in 18 rats were evaluated and summarized in Table 1. After re-exposure, there was no obvious adhesion. Blunt dissection was possible (grade 1 regarding to adhesion in all of cases) without any scar formation. 

**Fig. 1A-E:**
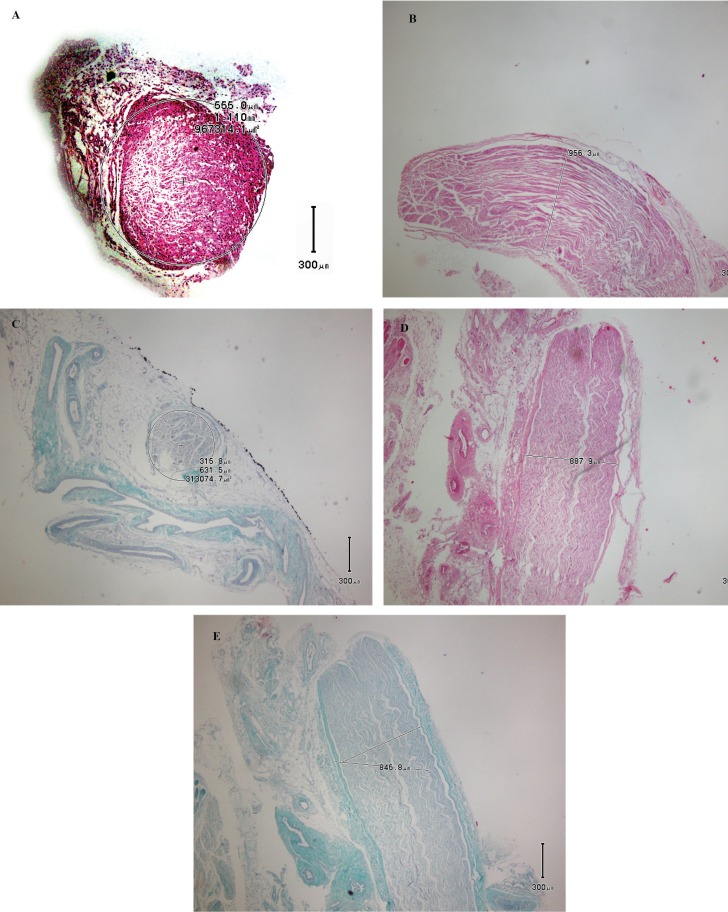
Silicone could significantly increase the myelin thickness in the side that silicone was applied

Silicone was significantly more effective in increasing myelin thickness in the side that silicone was applied) than the control side (Figure 1A-E). It was not associated with inflammation, scar formation, granuloma, and neuroma formation. No foreign body reaction occurred in liver, spleen and lungs after silicone application; but axonal regeneration did not improve with after its use.

**Table 1:** Histological data comparing control (right side) versus experimental (left side) nerves in repaired sciatic nerves in distal sectional analysis

**Variables**	**Group**		**P value**
	**Control**	**Experimental**	
Total fascicular area (mm^2^)	0.806 ±.57	0.894±0.57	0.6
Myelin fiber diameter (µm)	8.579±1.94	9.738±2.95	0.1
Axon diameter	4.352±1.33	3.716±0.96	0.1
Myelin thickness	4.167±1.77	6.249±2.78	0.01*
G-ratio	1.312±0.96	0.931±0.85	0.2
Connective tissue area (mm^2^)	0.747±0.68	0.827±0.81	0.5
Ratio connective tissue area/ fascicular area	1.210±1.06	1.166±1.12	0.9

* P<0.05: significant

There were no adhesion and inflammation in the site of repair. Trauma was not observed in gross observation. In addition, there were no documents in favor of trauma in microscopic evaluation. Axonal collapse and demyelination were not detected. 

## DISCUSSION

Scar formation due to accelerated response of fibroblasts poses a destructive role in peripheral nerve repair. Therefore, multiple investigations have been done for adjusting fibroblast activities and prevention scar formation.^5^^,^^6^^,^^8^^,^^9^^,^^11^^-^^13^^,^^18^^,^^22^ Numerous investigation have been designed on animal model for finding more effective materials as conduits for reconstructing nerves, so far.^7^^,^^9^^,^^12^^,^^23^^-^^25^ In this study, we evaluated silicon gel efficacy on rat sciatic nerve repair. Some of the previous investigations have supported useful effects of silicone on nerve reconstruction, whereas others have failed to show silicone advantages in comparison to other materials.^10^^,^^14^^,^^16^^,^^17^^,^^21^^,^^22^

Type 1 bovine collagen was used for sciatic nerve repair. This study was done on rats and showed that the rate of connective tissue and fascicular area/connective tissue ratio were significantly less than controls (p=0.001, p=0.001 respectively); while, axon regeneration was similar in both groups.^23^

This study showed that collagen wrapping in the site of reconstruction resulted into connective tissue reduction. Similarly, in our study silicone has not been effective in axon regeneration. However, results of our investigation showed that connective tissue area and fascicular area/connective tissue ratio did not have significant difference in right and left sciatic nerves. In addition, the significant increase in myelin thickness in the present study indicated that silicon may be effective in myelin repair and improving nervous function. More studies and molecular investigations on probable role of silicone on myelin production by Schwann cells are needed. 

In several studies, silicone has been compared with other materials for nerve reconstruction.^13^^,^^15^^-^^18^^,^^20^^-^^25^ For instance, Feng and colleagues evaluated nerve graft, silicone and chistosan-PLA for rat sciatic nerve reconstruction. They reported that nerve graft and chistosan-PLA were more effective than silicone. However, silicone has not had a destructive effect on nerve reconstruction in their study. In addition, inflammatory reaction was similar in all three materials.^10^

In another study, it was shown that lamotrigine could enhance the proliferation of Schwann cells and it played an important role in the regeneration of the injured sciatic nerve.^1^ In another research in 2005, application of silicone microelectrode on rat cortex resulted into a decrease in fiber density and cell body. However, the results of this study showed no disturbance in axon growth and myelinization.^7^

 In an animal study for sciatic nerve repair, damaged site was covered with silicone sheath. In this study, silicone was more effective than autonerve graft despite of a 5 mm gap.^1^^,^^10^ In addition, effectiveness of silicone gel has been established in the treatment of hypertrophic scars with scar elasticity and scar volume reduction.^16^^,^^21^

Adverse effects of silicone materials have been evaluated in several studies. Although no foreign body reaction was found in liver, spleen and lungs in our studied rats; but, some studies have shown severe inflammatory reaction and foreign body reaction by using silicone. For instance in a study, silicone prostheses in pig peritoneum foreign body reaction was seen.^21^ In another study foreign body reaction was reported in auxiliary lymph nodes after disruption of silicone implant in mammoplasty cases.^19^

There are some human investigations on techniques of nerve repair. In Lonborg study, silicone tube was used in ulnar nerve repair in 30 patients. In 5-year follow up, they observed that effects of silicone tube was similar to nerve graft.^17^

In another animal investigation, keratin extracted from human hair was applied as gel form in conduit. This study has shown that keratin gel application had a strong neuroinductive effect and was able to improve nervous function up to 30-100%.^6^

In summary, our study showed that silicone was significantly effective in increasing myelin thickness in peripheral nerves. It did not result into inflammation, scar, granuloma, and neuroma formation. Also, no foreign body reaction occurred in liver, spleen and lungs with silicone application; and axonal regeneration did not improve with using silicon. So, according to our study, it seems that silicone application in the cases with significant complications or in the cases that nerve graft is not possible would be an ideal option.

However, further studies with larger sample size are recommended for accurate evaluating effectiveness of silicone on myelin thickness. In addition, this is an investigation on rats and responses of silicone in human being may have some differences.

## CONFLICT OF INTEREST

The authors declare no conflict of interest. 

## Footenote

Please cite this paper as:

Fathi HR, Fathi M, Ghannadan AR, Alavion M, Kamyab K, Khazaipour Z, Amanpour S. The Healing Effectof Silicone Gel on Sciatic Nerve Injuries in Experimental Rat. World J Plast Surg 2014;3(2):93-98.
